# Working with Institutional Stakeholders: Propositions for Alternative Approaches to Community Engagement

**DOI:** 10.3390/ijerph16204010

**Published:** 2019-10-19

**Authors:** Jeffrey G. Cox, Minwoong Chung, Joseph A. Hamm, Adam Zwickle, Shannon M. Cruz, James W. Dearing

**Affiliations:** 1 Department of Communication Studies, Albion College, 611 East Porter Street, Albion, MI 49224, USA; 2 Department of Communication, Michigan State University, 404 Wilson Road, East Lansing, MI 48824-1212, USA; chungm12@msu.edu (M.C.); dearjim@msu.edu (J.W.D.); 3 School of Criminal Justice, Michigan State University, 665 Auditorium Road, East Lansing, MI 48824-1212, USA; jhamm@msu.edu (J.A.H.); zwicklea@msu.edu (A.Z.); 4 Environmental Science and Policy Program, Michigan State University, 293 Farm Lane, East Lansing, MI 48824-1212, USA; 5 Department of Communication Arts & Sciences, The Pennsylvania State University, University Park, PA 16802, USA; shannon.cruz@psu.edu

**Keywords:** community engagement, environmental communication, contamination, institutional stakeholders, remediation

## Abstract

Community engagement is a vital aspect of addressing environmental contamination and remediation. In the United States, the Superfund Research Program (SRP) forms groups of academic researchers from the social and physical sciences into Community Engagement Cores (CECs) and Research Translation Cores (RTCs), which focus on various aspects of informing and working with communities during and through the resolution of environmental crises. While this work typically involves engaging directly with members of affected communities, no two situations are the same. In some cases, alternative approaches to community engagement can be more appropriate for community improvement than traditional approaches. In particular, when research teams become involved in contamination crises at a late point in the process, their contributions can be better directed at supporting and reinforcing the work of institutional stakeholders charged with remediating pollution. Relevant factors include issue fatigue among a local population, and contamination that is due to a major employer. Supported by literature and experience, we offer several propositions that we believe lay out conditions that warrant such an approach by academic teams, rather than their direct engagement with unaffiliated individuals in communities.

## 1. Introduction

Community engagement is a multifaceted, important aspect of ensuring environmental health, especially when dealing with an environmental contamination crisis. Engagement processes can also take many forms. The one-way dissemination of translated information can help residents understand exposure vectors and health impacts. Interactive dialogue may assist community residents in resolving uncertainty and getting answers to pressing questions of their own. Citizen science allows community members to collect and provide data to researchers. Participatory action research enables community members to join in longitudinal, collaborative work with researchers, so that community members can better appreciate research assumptions and the limitations of scientific knowledge, while facilitating researchers’ better understanding of the values and interests that underlie community members’ opinions and behaviors. Environmental justice is commonly a central component of attempts at community engagement, since so many environmental contamination crises occur as a result of industrial processes that disproportionately affect low-income residents [1].

When an environmental contamination problem comes to light, any number of entities may be involved in communicating with a community, including local employers, elected officials and nonprofits, local-, state-, and federal-level government agencies, and university-based scientists. While each of these types of stakeholders may have pre-established protocols and plans for how to engage with affected communities, it is also the case that no two environmental contamination crises are the same. Each community-based problem has an interplay of factors that define it, including contaminants and environmental conditions, stakeholders involved, resident perceptions and concerns, and a community’s history and relationship to the responsible (polluting) party.

Environmental contamination crises are also dynamic. They change over time. Stakeholders learn and modify positions. They also come and go as testing, decision making, and remediation unfold, lawsuits are filed, and polluters go bankrupt or hang on. Natural environments change, too, not just as pollutants migrate through aquifers and soil, but also as soil, water, and air interact with contaminants. In some cases, there are surprising degrees of natural attenuation as the years and decades slip by. Change characterizes community concern, too. Newly discovered, a contamination problem often is met with fear and outrage, which can coalesce into mobilization. Later, after waiting for studies, assessments, and engineering reports, community interest and the willingness to engage can ebb. New crises arise, proponents of other issues demand attention, and the worry and practicing of new and inconvenient behaviors fades. Lessening community concern—regardless of whether contaminants are yet cleaned up—is also driven by the nature of preventive innovations. Community members may initially avoid contact with suspect soil, stop eating locally caught fish, and give up growing backyard vegetables. However, all these behavioral innovations to prevent or delay the onset of illness run into daily reinforced, observable evidence to the contrary. Grandparents and parents who adopt none of these precautions appear to be fine and are living into old age, while fishermen who eat what they catch are still out there on the river, unconcerned. As time passes, community concern dissipates and behavioral unpredictability settles in.

Community engagement can come from many sources. Given our background as academic researchers as part of a Superfund Research Program at a large state university, we focus our work in this manuscript on the role that university-based researchers can have in the community engagement process during environmental contamination and remediation. The Superfund Research Program is a program from the National Institute of Environmental Health Sciences (NIEHS) that funds research programs at individual US universities, called Superfund Research Programs (SRPs). Each of these university-based SRPs focuses on a specific set of environmental contamination problems—such as lead, arsenic, or dioxins—and conducts research to better understand how environmental contamination affects human and environmental health [2,3].

While most of these researchers are bench scientists studying the toxicological effects of environmental contaminants, many SRPs have dedicated research centers that focus on engaging with affected community members and translating academic research findings for public dissemination—termed Community Engagement Cores (CECs) and Research Translation Cores (RTCs) [4]. CECs generally focus on how the environmental contaminant that their SRP is researching has affected a local community that is facing a contamination problem, creating partnerships with local community groups and communicating research findings to community members. For example, our CEC at Michigan State University focuses on how dioxin contamination has affected members of a community in mid-Michigan, USA. Because our CEC (and others like it) comprises academic researchers from fields such as communication, environmental science, criminal justice, and human development, it has allowed us to bring a diversity of perspectives to bear on the community engagement process. Typically, because CECs and RTCs are focused on direct engagement with community members—in activities such as information dissemination and connecting residents with information sources—they are not as focused on supporting existing external change agents in these areas, such as the EPA [5].

## 2. What Kind of Engagement?

Community engagement and environmental communication can take multiple forms. Sometimes engagement efforts are focused on issues of sustainability and outreach, such as helping individuals use renewable energy. However, this manuscript focuses specifically on instances of community engagement that are meant to address environmental crises. There are multiple perspectives on the ways in which community engagement in time of crisis can and should be conducted, as well as the roles of external change agents and community-driven responses to environmental crises. Some of these discuss the merits of a bottom–up approach to community engagement, where public participation and deliberation are emphasized, along with equal say among stakeholders [6]. This is contrasted with a top-down approach, where “expert-led model[s] of development” dominate engagement processes [7]. In this manuscript, we do not discuss the relative (and undeniable) merits of either approach. Rather, we focus on situations in which a top-down approach, in the form of external change agent engagement with communities, is already ongoing.

The complexity and dynamic nature of both environmental contamination and affected communities suggests that community engagement as practiced by external change agents—federal and state agency staff and university-based centers and institutes—needs to be adaptive. The conditions that can characterize long-running environmental contamination problems suggest that there should be variability in who is engaged, how, to what extent, and when. In some cases, important contextual factors may necessitate a different kind of role for researchers who seek to engage communities—one that leverages their expertise in engagement and communication to help facilitate government agencies’ work, instead of directly engaging with community members.

In particular, these situations include those in which university researchers may become involved rather late in the ongoing contamination and engagement process. Institutional stakeholders, such as state- and federal- health and environmental agencies may already be far into their work of contamination remediation and engagement. Years or decades of news coverage of contamination may have served to blunt community members’ perceptions of threat and immediacy. This can lead to residents’ fatigue over issues’ coverage, in news media or local communications. A well-established company town culture may complicate the efforts of new, little-known external change agents to communicate risk information or engage with community members directly. In such situations where researchers join the process in the later stages—as opposed to right after an environmental disaster—a focus on supporting more established institutional stakeholders may allow for the expertise and resources of academic researchers to be used more effectively.

In this manuscript, we offer a set of propositions describing the conditions under which the efforts of university-based researchers may be more effective if—instead of focusing on more traditional, direct engagement activities aimed at members of the community—they focus on supporting institutional stakeholders that are involved in remediation. These propositions are drawn from both literature on engagement and community aspects of environmental contamination, as well as our own experiences in conducting research about community engagement across multiple studies. In so doing, we hope to provide readers with a literature-driven set of ideas that contextualizes our experiences. We offer an agenda for future research about the practice of community engagement in the context of environmental contamination, focusing on situations of late-stage involvement in which engagement with communities may be more beneficial if focused not on affected community members, but rather on those institutional stakeholders who work on their behalf.

## 3. Community Engagement as Commonly Practiced

The U.S. Centers for Disease Control and Prevention (CDC) defines *community engagement* as “the process of working collaboratively with and through groups of people affiliated by geographic proximity, special interest, or similar situations to address issues affecting the well-being of those people” [8]. Other government agencies in the U.S. offer similar definitions that emphasize the importance of partnerships and public participation [9,10]. In the environmental health literature, community engagement is often defined more simply as the building of relationships and partnerships directly with community members who are affected by hazardous environmental chemicals or that engage with researchers in the monitoring of environmental conditions [2,11,12]. Regardless of the domain, the ultimate goals of community engagement are typically establishing trust, mobilizing resources, enhancing communication, and as a result, raising the possibility of improved overall health outcomes for the engaged community [8,13,14,15]. Intermediate outcomes are often an increase in awareness of given environmental health issues and better understanding of behavioral precautions that individuals can take [3,16].

McCloskey et al. suggests five different types of community engagement that can be conceptualized along a continuum of increasing community involvement [15]. *Outreach* involves the lowest level of community involvement. Unlike the other types of community engagement, it features one-way communication from various stakeholders (e.g., external change agents and university-based researchers and staff) to community members. Thus, the primary outcomes of outreach are simply providing the community with information and establishing communication channels. *Consultation* requires more community involvement than outreach. The main objectives of this activity are gathering information/feedback from the community and developing connections. *Involvement* entails yet more direct interaction, such that the community is expected to actively participate in some of the activities. This allows both the outside change agents and the community to form a foundation for further partnerships. *Collaboration* features the community’s involvement on every aspect of the activities to address their issues. Throughout the collaboration, both parties establish trust and partnerships. *Shared leadership* entails the highest level of community engagement, such that the community has the right of the final decision making, based on the strong partnership and trust built throughout the previous community engagement activities.

## 4. Researchers and Institutional Stakeholders

Due to the complex and multi-faceted nature of environmental contamination problems, active collaboration between external change agents and the communities in which they are working is critical [17,18]. Social science researchers can play essential roles in this process, working to provide a comprehensive picture of the environmental, health, and social effects of contaminants on individuals in the affected community [19]. The role played by social scientists is often one of community-based participatory research (CBPR). CBPR has received much academic and practical attention since the mid-1990s, as the National Institute of Environmental Health Sciences (NIEHS) promoted the incorporation of social science in environmental research and practice [20]. The objective of social science in CBPR is to learn about the diverse impacts of environmental and health problems on community members by directly engaging with the affected communities [21]. Social scientists receive funding from government agencies to take part in CBPR and then are able to advance public health officials’ understanding of community residents’ concerns and values on the basis of research [19,20,22].

Social scientists also contribute to environmental health practices through transdisciplinary and translational work with institutional stakeholders [19,23,24,25,26,27]. For example, social scientists can lead government agencies’ intervention efforts, design more culturally and socially appropriate risk avoidance recommendations [19] and make scientific information more accessible to community members [22]. In addition, social science researchers can also help institutional stakeholders by developing or identifying evidence-based interventions, and pre-testing those interventions with the community in question before deployment [26].

While partnerships between institutional stakeholders and social scientists in instances of environmental health problems have become common [19], their work together has not been understood as community engagement. This, we believe, is an oversight both conceptually and practically. The centrality of external change agents in characterizing and ultimately remediating contamination, as well as the years-long dynamic nature of contaminants, stakeholders and communities, suggest that under certain conditions, research engagement with institutional stakeholders is more important and more appropriate than is research engagement with affected community members.

## 5. Propositions for Engagement with Institutional Stakeholders

We have identified five propositions that illuminate conditions that may complicate traditional orientations to community engagement and favor an approach of researchers working primarily with institutional stakeholders. These propositions arise from our own late involvement in contamination and remediation processes as part of the Michigan State University Superfund Research Program. Identification of these propositions was the result of a number of conversations as a Community Engagement Core. The earliest of these conversations were incidental, but as our relationships with the various agencies working in our study area deepened, the discussions became more intentional. Identification of the propositions reported here began as an effort by our lead author to organize our many discussions on the topic which was then shared among our research team and further developed into the current manuscript.

These propositions suggest areas for further research on the effectiveness of alternative forms of community engagement during environmental contamination and remediation. The first three propositions address late-stage community challenges that may significantly complicate traditional engagement processes, leading to recommendations for engagement with institutional stakeholders. The last two focus on institutional opportunities, highlighting elements and behaviors of the institutions themselves which may enhance the effectiveness of engaging directly with institutional partners. We argue that attending to these five propositions will help optimally position social scientists to positively affect the communities with which they work.

1 Direct (traditional) community engagement with unaffiliated community members is likely to be less effective when community members have issue fatigue about environmental contamination in their community.

Sometimes, the challenge is not that people are unaware of the environmental problems facing their community, but that they have been inundated with news coverage, public notices, government information dissemination, community forums, and citizen outrage about a problem for so long that they become effectively desensitized to further information on the subject [28]. The attention that community members give certain issues is finite and can reach a point of saturation. This *issue fatigue* results in a decreased level of attention and engagement from the public to the environmental problems that are still present in their communities. Issue fatigue is impacted by factors, both of the news media itself, and of individual cognitive processes within the audience. If the news media, locally or nationally, devote prolonged news coverage to a particular environmental issue, this can lead to a blunting of the perceived newsworthiness of their coverage [29]. Extensive news coverage of a particular environmental problem “ultimately reaches a point where sources are exhausted, news angles become worn and original (or mediated) events that can be thematically linked to the issue become short in supply, leading to a fading of news interest” [30] p. 501. However, it is not just a *staleness* or lack of novelty in the news that causes issue fatigue in residents, but a saturation of the topic itself, such that further continued coverage no longer represents “news”, since novelty is a fundamental attribute of topics that are considered worthy of news coverage [31,32].

On a cognitive level, an extended period of news coverage of an issue can lead individuals to disengage with issues that they have heard about time and again. This issue of desensitization is particularly heightened when the environmental problem is not a *hot crisis,* or one evoking a “sense of immediate and concrete risk with everyday relevance and strong emotional overtones,” but rather one that is slower moving or less noticeable [28]. Environmental health problems such as an oil spill or a disease epidemic can create a sense of urgency in affected communities, where individuals feel a heightened awareness of the problem and a resolve to counteract it; however, many environmental hazards affect community members in less immediate and tangible ways. For example, environmental contamination is often revealed over the course of years or decades, and many residents may not find out about these problems until long after the active contamination has ended. If community members have been exposed to these toxins for years without many visible health problems, they may feel a lack of urgency or needed reaction. An influx of additional risk information may be less likely to spur action and attention from the public if the problem seems to lack urgency, and therefore, an immediate response.

In a community with such issue fatigue challenges, the problem is less likely a lack of available information distribution and outreach. As a result, direct engagement efforts that seek to simply provide more information are unlikely to be especially impactful at this late stage in the process. This issue fatigue may even complicate efforts to engage communities in more collaborative co-management processes given that these efforts often rely on the willingness of participants to engage with each other and other stakeholders in identifying common ground regarding important aspects of the situation [15]. When issue fatigue leads community members to disengage, working directly with them can become especially difficult. Instead, research teams may be able to more effectively leverage their expertise and resources by engaging with relevant institutional stakeholders who are already active. One especially important opportunity arises when such stakeholders are in the process of remediating environmental contamination. Research teams can play crucial roles in supporting environmentally corrective work by lending expertise in addressing its environmental, biological, *and* social implications.

In our work in Midland, Michigan, we found a community that seemed largely disengaged from the environmental contamination in their area. Part of this was likely due to the long span of time that the contamination had been known to residents. Although remediation activities in the area started in 2012, the first national news reports about contamination occurred in the early 1980s. For many residents, this long period of hearing local and national news reports on the contamination could have led to a desensitization to the issues. Compounding these issues was the fact that many residents saw the contamination with a lack of concern, minimizing perceived dangers due to the belief that tangible health effects had not really been seen in the community [33].

2 Direct (traditional) community engagement with unaffiliated community members are complicated in communities where the organization responsible for the contamination is a major community employer.

A “company town” is a community in which a single corporation or other type of organization is the primary employer for large numbers of residents. Such companies often play a large cultural and economic role in the community, as well, such as funding schools, community centers, and other places that are central to a particular community’s identity [34]. In some company towns, large, community-defining employers have been in business for many decades and employed several generations of adults in families. Loyalty to them is strong. In this way, they go beyond simply being the area’s largest employer to being an inseparable pillar of the community. In some situations, a company town is affected by chemical contamination coming from the company itself. In such situations where a particular community is tied to, and reliant upon, a singular company that plays an outsized role in the contamination, the role of external change agents such as government agencies becomes much more complicated.

Perhaps unsurprisingly, a company town culture where the major employer is also the primary source of contamination can lead to a highly complex relationship between the company, community members, and institutional stakeholders who are focused on remediation processes. Individuals in the community may be reluctant to assign blame to a player that they see as the lifeblood of the community. In terms of environmental contamination, this blame can lead to a financial culpability for remediation. Residents may believe that the company’s positive influence on the community outweighs their culpability for the contamination, downplay the severity of their exposure, or even deny the company’s role in the problem. This can be compounded by the fact that many residents work for the company, causing further rejection of responsibility. In some cases, feelings of solidarity with the company’s status may lead to feelings of resentment for external change agents that they see as meddling in local affairs, or even attempting to impugn the reputation of an institution that is seen as vital to, and representative of, the community [35].

In such cases, efforts by university researchers to engage with or reach out to community members about the environmental problem may be met with apathy or even hostility [33]. Community members may see such efforts by university researchers as being disruptive activities by outsiders who have arrived at the situation late, and do not have a personal stake in the effects that remedial or regulative actions may have on the community.

We found such apprehension from local residents in Midland, where Dow Chemical is both the area’s largest employer and a major benefactor to the community. Many local parks and community spaces were funded by Dow. Additionally, one of the two local high schools is Herbert Henry Dow (the founder of Dow Chemical) High School. As a result of these strong ties, efforts to understand perceptions that Midland residents had towards the risks posed by the contamination were greatly complicated. While some residents gave thoughtful reflections of their attitudes, others expressed skepticism of Dow’s role and a defensiveness of one of the community’s largest contributors.

3 Direct engagement by academic teams with unaffiliated community members may be confusing if messages or inquiries from academic teams are contradictory or subvert an ongoing collaborative decision-process about remediation.

It is important for members of a social science research team to realize that their work with local communities has the potential to complicate or even undermine the work of agencies that are actively involved in the remediation process. Any efforts to engage with residents should be well coordinated with federal- and state-level government agencies involved in remediation processes, so as to not conflict with these agencies’ work. The potential for conflict occurs in the case of information sharing efforts where there is potential for the two sources of information to conflict, factually or ostensibly, if great care is not taken to coordinate across them. This regularly occurs, since scientific results and evidence-based information is necessarily probabilistic. Further, communities that are subject to numerous data gathering efforts can become tired of such activity [36], thereby reducing the likelihood of meaningful levels of participation in any of them. Finally, even when discrete groups are engaged, appropriate points of contact, institutional hierarchies, and overlap in the mandates, goals, and authority of these groups can foster a level of confusion that, at best, is frustrating, but at worst, leads community residents to lose faith in all of the groups working to help them, who may see them as monolithic. Extra care is therefore warranted when engagements are already underway, but especially when social scientists come into the situation relatively late (e.g., after the remediation process is already underway).

As we progressed in our work in Midland, we realized the importance of coordinating all of our work with our institutional stakeholders. Any time we wanted to collect data from residents about issues such as stigma [33] or risk perceptions [13], we made a point of discussing our proposed activities with the government agencies involved in the remediation process. In particular, we had regular conference calls with members of EPA, the Michigan Department of Environmental Quality, and the Michigan Department of Health and Human Services, the representatives of which were not always supportive of our plans. This enabled us to not only make sure that our proposed work was understood by these agencies and did not conflict with their own work, but also allowed us the opportunity to better understand their own recent activities in the area so that we could support rather than confuse residents of communities.

4 In times of environmental crisis, external change agents are centrally important to furthering community goals.

External change agents are important partners in dealing with communities that are facing large-scale challenges to health and welfare [17,18,37]. Local communities are typically not well equipped to be able to deal with large and complex chemical contamination by themselves, often lacking the funding, personnel, resources, and equipment needed; moreover, jurisdictional responsibilities can supersede local authorities. Because of this, institutional stakeholders from outside of the immediate area are often needed and necessarily involved in taking charge of remediation processes. Often, this will include a mixture of state and federal agencies focusing on environmental health. These agencies are able to use their expertise and resources to better address community needs, while bringing different strengths to the table. For example, federal agencies bring the experience and expertise of individuals who may have worked on contamination problems and remediation at different sites around the country.

Of course, external change agents pose challenges. By definition, they are outsiders to the immediate community they are entering. As such, they may need to take particular care to take into account community residents’ needs and concerns. This is one of the ways in which university-based researchers may be well positioned to help. These groups may take part in certain activities that can help the external change agents to do their work and better address community issues. For example, members of university-based teams can participate in community meetings that are mandated at EPA Superfund sites. By having a presence at these meetings and being in communication with relevant government agencies, these groups can play a useful role supporting change agents’ activities.

5 Engagement with institutional stakeholders calls for a diverse team and a transdisciplinary approach.

One of the distinct strengths of academic research and engagement teams is that they can draw upon the diversity of fields represented in a university. Unlike institutional stakeholders involved in remediation processes, whose members’ bench science training may be specialized and homogenous, social scientists can represent a variety of backgrounds. This educational, theoretical, and methodological diversity means that they can leverage a wide range of experiences and training into supporting institutional stakeholders [38]. Physical and environmental scientists can ensure that the team understands the bench science of the remediation work, helping the team with their engagement. The addition of social science and communication researchers can enable institutional stakeholders to better understand their work from a social perspective [19]. For example, social scientists can create surveys to gain an understanding of the behaviors and perceptions of community members, which will help inform the work of agencies involved in the remediation. Communication scientists can be involved in activities such as assessing the readability of written works from the agencies, and both take part in and assess community meetings.

Our own work has benefitted from having a diverse set of backgrounds among our members, including faculty and graduate students from fields such as environmental science and policy, criminal justice, human development, and communication. Not only did this allow us to have a better understanding of the work that the agencies involved in remediation were doing, but it allowed us to conduct social science research that was scientifically informed and relevant to the environmental issues at hand. Our work with institutional representatives was greatly helped by our ability to bring complementary and supplementary strengths to the process.

## 6. Conclusions

While traditional orientations to community engagement are an important part of any situation involving environmental contamination and remediation, different contexts may require different engagement approaches. In particular, when research teams become involved in a situation of environmental contamination and remediation at a rather late stage in the process, the usefulness of their contributions may change. The conditions described in this manuscript dictate that an approach to community engagement focusing on direct engagement with local affected community members may be less effective than one that aims to support institutional stakeholders that are already actively engaged. University-based teams bring resources and expertise to the responsibilities of institutional stakeholders that can improve communication with, and environmental and health outcomes in, communities.

However, additional questions remain that are ripe for future research and theorizing on community engagement and environmental communication. For example, what should the ultimate goals be of academic researchers involved in environmental crises more generally, and what factors influence their ability to accomplish these goals? Should they simply be focused on disseminating information on environmental risk, or is there a more active role that researchers can take? Systematic attention to these questions would help researchers working in communities where these issues are more established and would provide researchers with much needed information from which to make difficult decisions like deciding whether to initiate a new program of direct engagement in communities where the official response neglects or even malevolently affects disenfranchised residents.

One especially useful line of inquiry would focus on how university-based researchers can leverage the unique diversity of experiences, perspectives, and disciplinary backgrounds that exist in their educational institutions. To be clear, the foundational “to what end?” question implicates values in a way that will always require individual teams to use their best judgment, but systematic evaluation of these ancillary questions of how university resources can best be integrated and deployed are well within the realm of science and do impact the roles that these teams can reasonably accomplish. Academic research teams can and should draw upon the wide range of expertise at their disposal—exploring research questions driven by work in the physical sciences, social sciences, and humanities. Understanding how this confluence of perspectives plays out in reality, and the extent to which inter- and transdisciplinary research help government agencies and other institutional stakeholders effectively engage members of an affected community, matters. Systematic attention to these issues could, for example, shed light on how researchers with diverse backgrounds can assist in activities like designing flyers, facilitating community forums, and setting priorities for deliberative discussions. Or, in what ways could researchers with backgrounds in sociology, criminal justice, and environmental science help government agencies to understand issues like institutional trust and environmental justice? Academic researchers with backgrounds in the social sciences and humanities can be useful for helping government agencies understand the important and complex social and political intricacies of the communities in which they are working. Community engagement is a complex, context-dependent process, and one for which research teams with a variety of perspectives and experiences are particularly well suited.

## References

[1] Environmental Protection Agency (2006). Brownfields Fact Sheet: Solid Waste and Emergency Response.

[2] Landrigan P.J., Wright R.O., Cordero J.F., Eaton D.L., Goldstein B.D., Hennig B., Maier R.M., Ozonoff D.M., Smith M.T., Tukey R.H. (2015). The NIEHS Superfund Research Program: 25 years of translational research for public health. Environ. Health Perspect..

[3] National Institute of Environmental Health Sciences (2019). Superfund Research Program. https://www.niehs.nih.gov/research/supported/centers/srp/index.cfm.

[4] National Institute of Environmental Health Sciences (2011). Community Engagement and Research Translation. https://www.niehs.nih.gov/research/supported/centers/srp/outreach/index.cfm.

[5] National Institute of Environmental Health Sciences (2019). Community Engagement Cores. https://www.niehs.nih.gov/research/supported/centers/core/coe/index.cfm.

[6] Fraser E.D., Dougill A.J., Mabee W.E., Reed M., McAlpine P. (2006). Bottom up and top down: Analysis of participatory processes for sustainability indicator identification as a pathway to community empowerment and sustainable environmental management. J. Environ. Manag..

[7] Tandon R. (2008). Participation, citizenship and democracy: Reflections on 25 years of PRIA. Community Dev. J..

[8] Centers for Disease Control and Prevention (1997). Principles of Community Engagement.

[9] National Institute of Environmental Health Sciences (2012). Partnerships for Environmental Public Health: Evaluation Metrics Manual.

[10] Charnley S., Engelbert B. (2005). Evaluating public participation in environmental decision-making: EPA’s superfund community involvement program. J. Environ. Manag..

[11] Pezzoli K., Tukey R., Sarabia H., Zaslavsky I., Miranda M.L., Suk W.A., Lin A., Ellisman M. (2007). The NIEHS environmental health sciences data resource portal: Placing advanced technologies in service to vulnerable communities. Environ. Health Perspect..

[12] McElfish J., Pendergrass J., Fox T. (2016). Clearing the Path: Citizen Science and Public Decision Making in the United States.

[13] Hamm J.A., Cox J., Zwickle A., Zhuang J., Cruz S., Upham B.L., Chung M., Dearing J.W. (2018). Trust in whom? Dioxin, organizations, risk perception, and fish consumption in Michigan’s Saginaw Bay watershed. J. Risk Res..

[14] Christopher S., Watts V., McCormick A., Young S. (2008). Building and maintaining trust in a community-based participatory research partnership. Am. J. Public Health.

[15] McCloskey D.J., Aguilar-Gaxiola S., Michener J.L., Akintobi T.H., Bonham A., Cook J., Coyne-Beasley T., Dozier A., Duffy R., Eder M. (2011). Principles of Community Engagement.

[16] Finn S., Collman G. (2016). The pivotal role of the social sciences in environmental health sciences research. New Solut..

[17] Bryson J.M., Crosby B.C., Stone M.M. (2006). The design and implementation of Cross-Sector collaborations: Propositions from the literature. Public Adm. Rev..

[18] Institute of Medicine (2002). The Future of the Public’s Health in the 21st Century.

[19] Hoover E., Renauld M., Edelstein M.R., Brown P. (2015). Social science collaboration with environmental health. Environ. Health Perspect..

[20] O’Fallon L.R., Dearry A. (2002). Community-based participatory research as a tool to advance environmental health sciences. Environ. Health Perspect..

[21] Israel B.A., Schulz A.J., Parker E.A., Becker A.B. (1998). Review of community-based research: Assessing partnership approaches to improve public health. Annu. Rev. Public Health.

[22] English P.B., Olmedo L., Bejarano E., Lugo H., Murillo E., Seto E., Wong M., King G., Wilkie A., Meltzer D. (2017). The Imperial County community air monitoring network: A model for community-based environmental monitoring for public health action. Environ. Health Perspect..

[23] Bryson J.M., Crosby B.C., Stone M.M. (2015). Designing and implementing cross-sector collaborations: Needed and challenging. Public Adm. Rev..

[24] Buick F., Blackman D., O’Flynn J., O’Donnell M., West D. (2016). Effective practitioner-scholar relationships: Lessons from a coproduction partnership. Public Adm. Rev..

[25] Franklin A.L., Grossman A., Le J., Shafer M. (2019). Creating broader research impacts through boundary organizations. Public Adm. Rev..

[26] Green L.W., Mercer S.L. (2001). Can public health researchers and agencies reconcile the push from funding bodies and the pull from communities?. Am. J. Public Health.

[27] Lambright W.H. (2008). Government and science: A troubled, critical relationship and what can be done about it. Public Adm. Rev..

[28] Ungar S. (2000). Knowledge, ignorance and the popular culture: Climate change versus the ozone hole. Public Underst. Sci..

[29] Vasterman PL M. (2005). Media-hype: Self-reinforcing news waves, journalistic standards and the construction of social problems. Eur. J. Commun..

[30] Djerf-Pierre M. (2012). The crowding-out effect. J. Stud..

[31] Downs A. (1972). Up and down with ecology—The “issue-attention cycle”. Natl. Aff..

[32] Dearing J.W., Rogers E.M. (1996). Agenda-Setting.

[33] Zhuang J., Cox J.G., Cruz S., Dearing J.W., Hamm J.A., Upham B. (2016). Environmental stigma: Resident responses to living in a contaminated area. Am. Behav. Sci..

[34] Neumann P. (2016). Toxic talk and collective (in)action in a company town: The case of La Oroya, Peru. Soc. Probl..

[35] Solecki W.D. (1996). Paternalism, pollution and protest in a company town. Political Geogr..

[36] Carrera J.S., Key K., Bailey S., Hamm J.A., Cuthbertson C.A., Lewis E.Y., Woolford S.J., DeLoney E.H., Greene-Moton E., Wallace K. (2019). Community science as a pathway for resilience in response to a public health crisis in Flint, Michigan. Soc. Sci..

[37] Rogers E.M. (2003). Diffusion of Innovations.

[38] Frabutt J.M. (2010). Supporting community safety through university-community partnerships: Exploring models of engagement. J. Community Engagem. High. Educ..

